# *MAP3K1* Variant Causes Hyperactivation of Wnt4/β-Catenin/FOXL2 Signaling Contributing to 46,XY Disorders/Differences of Sex Development

**DOI:** 10.3389/fgene.2022.736988

**Published:** 2022-03-03

**Authors:** Hong Chen, Qingqing Chen, Yilin Zhu, Ke Yuan, Huizhu Li, Bingtao Zhang, Zexiao Jia, Hui Zhou, Mingjie Fan, Yue Qiu, Qianqian Zhuang, Zhaoying Lei, Mengyao Li, Wendong Huang, Li Liang, Qingfeng Yan, Chunlin Wang

**Affiliations:** ^1^ Department of Pediatrics, The First Affiliated Hospital of Zhejiang University School of Medicine, Hangzhou, China; ^2^ Fuzhou Children’s Hospital of Fujian Medical University, Fuzhou, China; ^3^ Department of Pediatrics, Lishui City People’s Hospital, Lishui, China; ^4^ College of Life Sciences, Zhejiang University, Hangzhou, China; ^5^ Department of Diabetes Complications and Metabolism, The Beckman Research Institute, City of Hope National Medical Center, Duarte, CA, United States; ^6^ Key Laboratory for Cell and Gene Engineering of Zhejiang Province, Hangzhou, China

**Keywords:** disorders of sex development, MAP3K1 variant, Wnt4/β-catenin signaling, FOXL2, DMRT1

## Abstract

**Background:** 46,XY disorders/differences of sex development (46,XY DSD) are congenital conditions that result from abnormal gonadal development (gonadal dysgenesis) or abnormalities in androgen synthesis or action. During early embryonic development, several genes are involved in regulating the initiation and maintenance of testicular or ovarian-specific pathways. Recent reports have shown that *MAP3K1* genes mediate the development of the 46,XY DSD, which present as complete or partial gonadal dysgenesis. Previous functional studies have demonstrated that some *MAP3K1* variants result in the gain of protein function. However, data on possible mechanisms of *MAP3K1* genes in modulating protein functions remain scant.

**Methods:** This study identified a Han Chinese family with the 46,XY DSD. To assess the history and clinical manifestations for the 46,XY DSD patients, the physical, operational, ultra-sonographical, pathological, and other examinations were performed for family members. Variant analysis was conducted using both trio whole-exome sequencing (trio WES) and Sanger sequencing. On the other hand, we generated transiently transfected testicular teratoma cells (NT2/D1) and ovary-derived granular cells (KGN), with mutant or wild-type *MAP3K1* gene. We then performed functional assays such as determination of steady-state levels of gender related factors, protein interaction and luciferase assay system.

**Results:** Two affected siblings were diagnosed with 46,XY DSD. Our analysis showed a missense c.556A > G/p.R186G variant in the *MAP3K1* gene. Functional assays demonstrated that the MAP3K1^R186G^ variant was associated with significantly decreased affinity to ubiquitin (Ub; 43–49%) and increased affinity to RhoA, which was 3.19 ± 0.18 fold, compared to MAP3K1. The MAP3K1^R186G^ led to hyperphosphorylation of p38 and GSK3β, and promoted hyperactivation of the Wnt4/β-catenin signaling. In addition, there was increased recruitment of β-catenin into the nucleus, which enhanced the expression of pro-ovarian transcription factor *FOXL2* gene, thus contributing to the 46,XY DSD.

**Conclusion:** Our study identified a missense *MAP3K1* variant associated with 46,XY DSD. We demonstrated that MAP3K1^R186G^ variant enhances binding to the RhoA and improves its own stability, resulting in the activation of the Wnt4/β-catenin/FOXL2 pathway. Taken together, these findings provide novel insights into the molecular mechanisms of 46,XY DSD and promotes better clinical evaluation.

## Introduction

Disorders/differences of sex development (DSD) are a set of congenital conditions, characterized by atypical development in chromosomal, gonadal, or other anatomical sex characteristics (Lee et al., 2006; Ono and Harley, 2013). Previous definitions and terminologies related to DSD have raised controversies on the exact conditions and incidence of DSD (Lee et al., 2006). Besides, the overall incidence of genitally ambiguous patients at birth remains undefined. Nevertheless, it has been reported that the incidence of DSD could span between 0.33 and 1.7% of all congenital reproductive organ abnormalities (Blackless et al., 2000; Nordenvall et al., 2014). 46,XY DSD encompass a wide category of phenotypes such as defective formation of gonads or gonadal dysgenesis, defects in androgen synthesis or action, while some may have normal testes with 5α reductase deficiency. DSDs significantly affect the patients’ physical and social-psychological health, and increases financial burden in the society. Although whole-exome sequencing has become the mainstay clinical test for analysis of known or novel genes associated with DSD, only 13–20% of DSD patients are estimated to have an accurate clinical genetic diagnosis (Lee et al., 2006; Eggers et al., 2016). Out of the total tests, only 35–43% resulted in identification of a specific pathogenic gene (Baxter et al., 2015; Eggers et al., 2016), suggesting that the causes of 46,XY DSD remain undefined. Accurate genetic diagnosis of the 46,XY DSD patients remains crucial for early treatment and prediction of associated risks of malignant tumors.

Although functions of many sex-linked genes are relatively defined, there is increased discovery of new genes with specific roles while the roles of other genes that mediate gonad developmental pathways remain elusive. Recent studies have shown that variants of the human mitogen-activated protein kinase 1 (encoded by *MAP3K1* gene, OMIM*600,982) are relatively common and are attributed to ∼18% of the 46,XY DSD cases (OMIM#613762) (Eggers et al., 2016). These *MAP3K1* variants were first discovered in two large families with 46,XY DSD and were characterized by autosomal dominant inheritance and sex-restricted inheritance patterns (Pearlman et al., 2010). Clinical manifestations of 46,XY individuals with the pathogenic *MAP3K1* gene variants range from severe DSD (with or without gonadoblastoma) to milder phenotypes such as hypospadias, cryptorchidism, and a small penis (Pearlman et al., 2010; Ostrer, 2014; Eggers et al., 2016; Granados et al., 2017; Igarashi et al., 2020; Cheng et al., 2021). However, data on the 46,XY DSD as a result of the *MAP3K1* gene variants remain relatively limited. Besides, related genotype-phenotype correlations require functional and clinical analysis, which include age, genital phenotype, intra-family phenotypic variation, and gonadal histology (Pyle and Nathanson, 2017).

In this study, we analyzed clinical features, laboratory results, and ultra-sonographical data from two siblings with 46,XY DSD. Gene sequencing data revealed a missense variant in the *MAP3K1* gene c.556A > G (p.R186G). In addition, to elucidate pathogenic mechanisms associated with the *MAP3K1* variant, we performed a series of functional assays.

## Materials and Methods

### Subjects

This study was approved by the Ethics Committee of The First Affiliated Hospital, Zhejiang University School of Medicine, China (approval number 2018–727). Written and informed consent was obtained from the parents of the proband.

### Clinical Evaluations

The proband and her elder sister were assessed for medical history and clinical manifestations. Physical examination, especially on the external genitalia, was also performed. Thereafter, laboratory examination including hormonal analysis (follicle-stimulating hormone, luteinizing hormone, estradiol, progesterone, prolactin and testosterone), karyotype analysis, imagological examination, laparoscopy and gonadal biopsy with histopathological examination were performed.

### Cytogenetic and Molecular Studies

Karyotype analysis was carried out using standard techniques. Briefly, genomic DNA was obtained from both the proband and her parents via peripheral blood leukocytes using standard procedures. We then performed whole-exon sequencing (WES) as previously described (Chen et al., 2019). The Sanger sequencing variant detected in the *MAP3K1* gene was described according to the NCBI entry NG_031884.1 (NM_005921.2). The variants were named according to the Human Genome Variation Society (HGVS) sequence variant nomenclature (den Dunnen et al., 2016).

### Conservation and Pathogenicity Analysis of the Variant

We downloaded various MAP3K1 protein sequences from Uniprot (https://www.uniprot.org/), while ClustalX program (http://ftp-igbmc.u-strasbg.fr/pub/ClustalX/) was used for sequence alignment. Sequence alignment results were displayed online using ConSurf (https://consurf.tau.ac.il/) (Ashkenazy et al., 2010; Ashkenazy et al., 2016).

The variant’s pathogenicity was analysed using the webserver; PREDICT-SNP (https://loschmidt.chemi.muni.cz/predictsnp1/) (Bendl et al., 2014). This tool employs a consensus of five different predictors, which include PredictSNP, PolyPhen-1 (Ramensky et al., 2002), PolyPhen-2 (Adzhubei et al., 2010), SIFT (Ng and Henikoff, 2003), and SNAP (Ramensky et al., 2002) along with provision of a confidence score for the predictions.

### Homologous Modeling of Human MAP3K1 Protein

Due to lack of experimental structure or sufficient homology models, the 3D structure of MAP3K1 protein was generated via ab initio modeling. The amino acid sequence of wild-type human MAP3K1 protein was retrieved from UniProt database (http://www.uniprot.org). The 3D structure of the wild-type human MAP3K1 protein was predicted through Iterative Threading ASSEmbly Refinement (I-TASSER) (https://zhanglab.ccmb.med.umich.edu/I-TASSER/) (Roy et al., 2010; Yang et al., 2015). Thereafter, the online server Dynamut (http://biosig.unimelb.edu.au/dynamut/) was used to assess local conformation of wild-type and variant proteins, and assess changes, if any, in protein flexibility (Rodrigues et al., 2018). A structural representation was generated using PyMOL 2.4 (https://pymol.org/2/), a molecular visualization system in the open-source foundation.

### Plasmid Construction

Wild-type full-length human *MAP3K1* complementary DNA (cDNA) (NM_005921.2) was chemically synthesized and cloned into a pCDH vector. The c.556A > G variation was introduced into the *MAP3K1* sequence using an In-Fusion HD Cloning kit (TaKaRa, United States). Forward and reverse primers used for PCR amplification were 5′ CGT​CCA​GAG​GAA​CGA​ATG​ATC​GGG​GAG​AAA​CTG​AAG​GCA​A3′ and 5′ GAT​CAT​TCG​TTC​CTC​TGG​ACG​ATC​ATC​CA3’, respectively. We then constructed plasmids transiently expressing *MAP3K1*-3×Flag, *MAP3K1*-3×HA, *RHOA*-3×Flag, or *UB*-3×Ha were for co-immunoprecipitation assays. The *MAP3K1* gene was cloned into the plasmid p3×Flag-CMV-14 or pcDNA3.1(+)-3×HA plasmid. *RHOA* (NM_001664) gene was cloned into the p3×Flag-CMV-14 plasmid, while *UB* (NM_021009.7) gene was cloned into the pcDNA3.1(+)-3×HA plasmid. The ligated product was transformed into *Escherichia coli* (DH5α cells). The recombinant plasmid clones were sequenced to verify the integrity of the insertion sequences.

### TCF/LEF Luciferase Assay

HEK-293T cells were co-transfected with TCF/LEF-TOP flash reporter plasmid and wild-type or variant *MAP3K1* gene expression vectors. The cell transfection experiments were performed in a *24-well* plate. The plasmids were transfected into HEK-293T cells using Lipofectamine 3,000 (Thermo Fisher, United States), according to the manufacturer’s instructions. After each variant was co-transfected, the DNA ratio of the *MAP3K1* and TCF/LEF-TOP flash reporter plasmids (Genechem, China) were 1:1 (250 ng each). After transfection, we employed the luciferase Reporter Assay System (Promega, United States) at 24 h, 36 h, 48 h, 60 h, and 72 h. The transfection efficiency was quantitatively assessed using quantitative PCR (qPCR), as shown in Supplementary Figure S4. The experiments were independently repeated at least five times, with at least three replicates. Data were expressed as mean ± SEM and analyzed using a *t*-test.

### Measurement of Protein Interaction in Cells

The structural complementation reporter system, NanoLuc^®^ Binary Technology (NanoBiT) (Promega, Cat#N2014), was used to analyze protein interactions. The NanoBiT consists of Large BiT (LgBiT; 18 kDa) subunit and a small complimentary peptide (SmBiT; 3.6 kDa) (Dixon et al., 2016). When the two target proteins interact, the LgBiT and SmBiT subunits converge to form an active enzyme, which produces a bright luminescent signal in the presence of a substrate. For NanoBiT protein interaction experiments, the *MAP3K1* gene CDs and the N-terminus of the LgBiT or the C-terminus of the SmBiT were fused to form *MAP3K1*-LgBiT-N or *MAP3K1*-SmBiT-C plasmids, respectively. The *RHOA* gene and the N-terminus of the LgBiT were fused to form *RHOA*-LgBiT-N expression plasmid. On the other hand, the *UB* gene and the C-terminus of the LgBiT were fused to form the *UB-*LgBiT-C expression plasmid. HEK293T cells were then co-transfected with *MAP3K1*-SmBiT-C and *RHOA*-LgBiT-N to assess the ability of the MAP3K1-WT or MAP3K1-Mut to interact with RhoA. Similarly, the HEK293T cells were co-transfected with SmBiT-*MAP3K1*-N and LgBiT*-UB*-C to evaluate the ability of the MAP3K1-WT or MAP3K1-Mut to interact with Ub. The HEK-293T cells in a *24-well* plate were transfected with a total of 500 ng DNA using a Lipofectamine™ 3,000 transfection reagent. Twenty-4 hours after transfection, the culture medium was replaced with fresh OPTI-MEM, and then a diluted substrate was directly added to each well. Fluorescence intensity was measured using a Multimode Plate Reader VICTORNivo (ND-1000, Thermo Fisher). The transfection efficiency was quantitatively analyzed through qPCR, as shown in Supplementary Figure S5. The experiments were independently repeated at least three times, with at least three replicates. Data were expressed as mean ± SEM and analyzed using a *t*-test.

### Co-Immunoprecipitation Assay

Co-IP was performed as previously described (Wang et al., 2019). Briefly, the *MAP3K1*-3×HA was co-transfected with *RHOA*-3×Flag, while *MAP3K1*-3×Flag was co-transfected with *UB*-3×HA plasmid using Lipofectamine 2000. At 48h post-transfection, the cells were collected and washed with PBS, and then harvested in ice-cold modified RIPA lysis buffer. Cell lysates were immunoprecipitated, and then incubated overnight with anti-Flag or anti-HA magnetic beads (GenScript, China) at 4°C. The proteins were denatured at 95°C for 10 min and then resolved in SDS-PAGE. Thereafter, the blots underwent western blot analysis with anti-HA or anti-FLAG antibodies. Protein band intensities were determined and used to calculate the relative ratio of Co-IP to IP signal. Data were shown as mean ± SEM (*n* = 3) and analyzed using Mann-Whitney *U* test.

### Cell Culture

The HEK293T cells and a human pluripotent teratocarcinoma testicular origin malignant embryonal carcinoma cell line (NT2/D1, ATCC^®^ CRL-1973™) were cultured in HG-DMEM (Invitrogen, United States), containing 10% fetal bovine serum (FBS). On the other hand, the ovarian origin granulosa cell tumor line (KGN, RIKEN Cell Bank^®^ RCB1154) was cultured in DMEM/F12 medium (Invitrogen, United States), containing 10% FBS. The cells were grown at 37°C with 5% CO_2_.

### Quantitative Real-Time Polymerase Chain Reaction

Total RNA was obtained using Total RNA Extraction Reagent (Promega). Thereafter, mRNA expression levels were analyzed by real-time PCR using the ABI PRISM 7900HT Sequence Detection System (Applied Biosystems) (Liu et al., 2014). Expression values were normalized using β-actin (*ACTB* gene). Supplementary Table S1 shows the basic information on primers, gene name, gene ID and NCBI reference sequence.

### Western Blot Analysis

The cells were collected and lysed in RIPA lysis buffer. Equal amount of protein (25 ug), as quantitated using the BCA Protein Assay, from each group was resolved in 10% SDS-PAGE and then electro-transferred onto a polyvinylidene difluoride membranes for western blott analysis (Li et al., 2018). The sources and catalog numbers of the antibodies for Western blotting are as listed in Supplementary Table S2. Each experiment was independently performed at least three times.

### Statistical Analysis

Western blot data were processed using the Fiji/ImageJ software. Statistical analyses were performed in Graph Pad Prism 7 program. Statistical significance was tested using a *t*-test or a Mann-Whitney *U* test. The results were drawn from at least three independent experiments. Data were shown as mean ± SD (*n* ≥ 3). * indicates *p* < 0.05, ** indicate *p* < 0.01 and *** indicate *p* < 0.001.

## Results

### Clinical Evaluation

**FIGURE 1:**
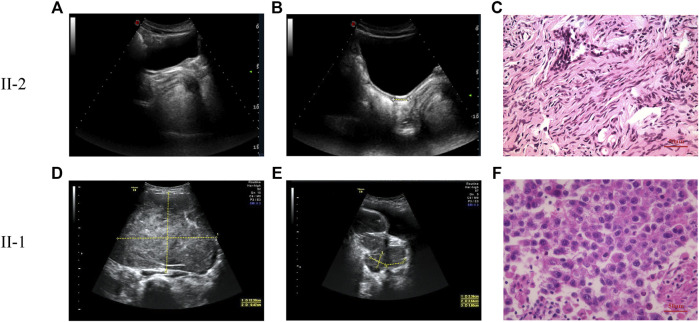
The pelvic ultrasound and pathological images of patients. **(A,B)** The pelvic ultrasound of proband (II-2). Yellow dashed lines indicate the length of ultrasonography. **(A)** No identifiable testicular or ovarian tissue was observed in the pelvic ultrasound. **(B)** Underdeveloped uterus (1.5 × 0.5 × 1.0 cm) of the proband. **(C)** Pathological section analysis of proband (HE, ×40). Pathological evaluation reveals that the bilateral streak gonads are ovarian interstitial-like cells and germinal epithelium, without any malignant transformation. **(D,E)** The pelvic ultrasound of patient 2 (II-1). Yellow dashed lines indicate the length of ultrasonography. **(D)** Left ovarian mass (12.2 × 9.5 × 7.0 cm). **(E)** Right ovarian mass (2.3 × 2.6 × 1.8 cm). **(F)** Pathological section analysis of patient II-1 (HE, ×40). The pathological evaluation identified the bilateral ovarian tumors as dysgerminoma.

A 13.5-year-old girl was admitted to the First Affiliated Hospital of Zhejiang University School of Medicine due to lack of breast development and menstruation. Both parents were Han Chinese, healthy and nonconsanguineous. Her height and weight were 157.8 cm (+0.03SDS) and 39 kg (-1.06SDS), respectively. Based on the Tanner scale, her breasts and pubic hair were Tanner I. External genitalia presented as a normal female with no clitoromegaly. Ultrasonography analysis revealed a rudimentary uterus (1.5 × 0.5 × 1.0 cm) and no identifiable gonadal tissue (Figures 1A,B). Analysis of the blood hormonal features of the proband demonstrated a sex steroid deficiency and high gonadotropin levels (Table 1). Further diagnostic tests demonstrated a 46,XY karyotype. Following genetic testing, the proband underwent a laparoscopic bilateral gonadectomy. In addition, stunted uterine-like tissue was observed during the operation, but no obvious abnormalities were found in the bilateral fallopian tubes. Streak gonads could be seen below the bilateral fallopian tubes. Pathological evaluation revealed that the bilateral streak gonads were ovarian interstitial-like cells and germinal epithelium, without any malignant transformation (Figure 1C).

**TABLE 1:** Clinical data of patients.

Items	Patients		
	Proband	II-1	
Age at diagnosis	13.3-year-old	14-year-old	
Karyotype	46,XY	46,XY	
Phenotype	Normal female external genitalia, a blind vagina and rudimentary uterus	Normal female external genitalia, a blind vagina and bilateral ovarian dysgerminoma	
Blood hormonal characteristics			Normal value
FSH	107.10**↑**	112.41↑	1.37–6.97 IU/L
LH	32.89**↑**	65.76↑	0.33–6.10 IU/L
Estradiol	＜43.31**↓**	＜43.31**↓**	136.76–315.00 pmol/L (female)
Progesterone	1.46	4.83↑	1.27–3.82 nmol/L
PRL	414.884	610.56	38.16–619.04 mIU/L
Testosterone	0.312**↓**	2.08↑	0.70–1.70 nmol/L

FSH: follicle-stimulating hormone; LH: luteinizing hormone; PRL: prolactin.

Her elder sister, a 19-year-old female, had undergone a laparoscopic bilateral ovarian tumor removal 5 years ago. Her external genitalia presented as a normal female with no clitoromegaly. The vagina ended in a blind pouch, with normal labia. However, the vagina and urethra had separate openings. Her karyotype was also 46,XY. Ovarian ultrasonography indicated the presence of a 12.2 × 9.5 × 7.0 cm mass in the left ovary and a 2.3 × 2.64 × 1.8 cm mass in the right ovary (Figures 1D,E). Surgical records showed that she had bilateral ovarian tumors of about 20.0 × 14.0 × 7.0 cm on the left and 5.0 × 2.5 × 2.0 cm on the right. In addition, HE staining, placental alkaline phosphatase staining, and immunohistochemical staining (CD117) classified her bilateral ovarian tumor as dysgerminoma (Figure 1F, Supplementary Figure S1). Serum gonadotropin levels of the sister were elevated, and gonadal steroid levels were low, demonstrating gonadal failure. Based on the above clinical characteristics, both patients were diagnosed with 46,XY DSD.

### Genetic Diagnosis

**FIGURE 2:**
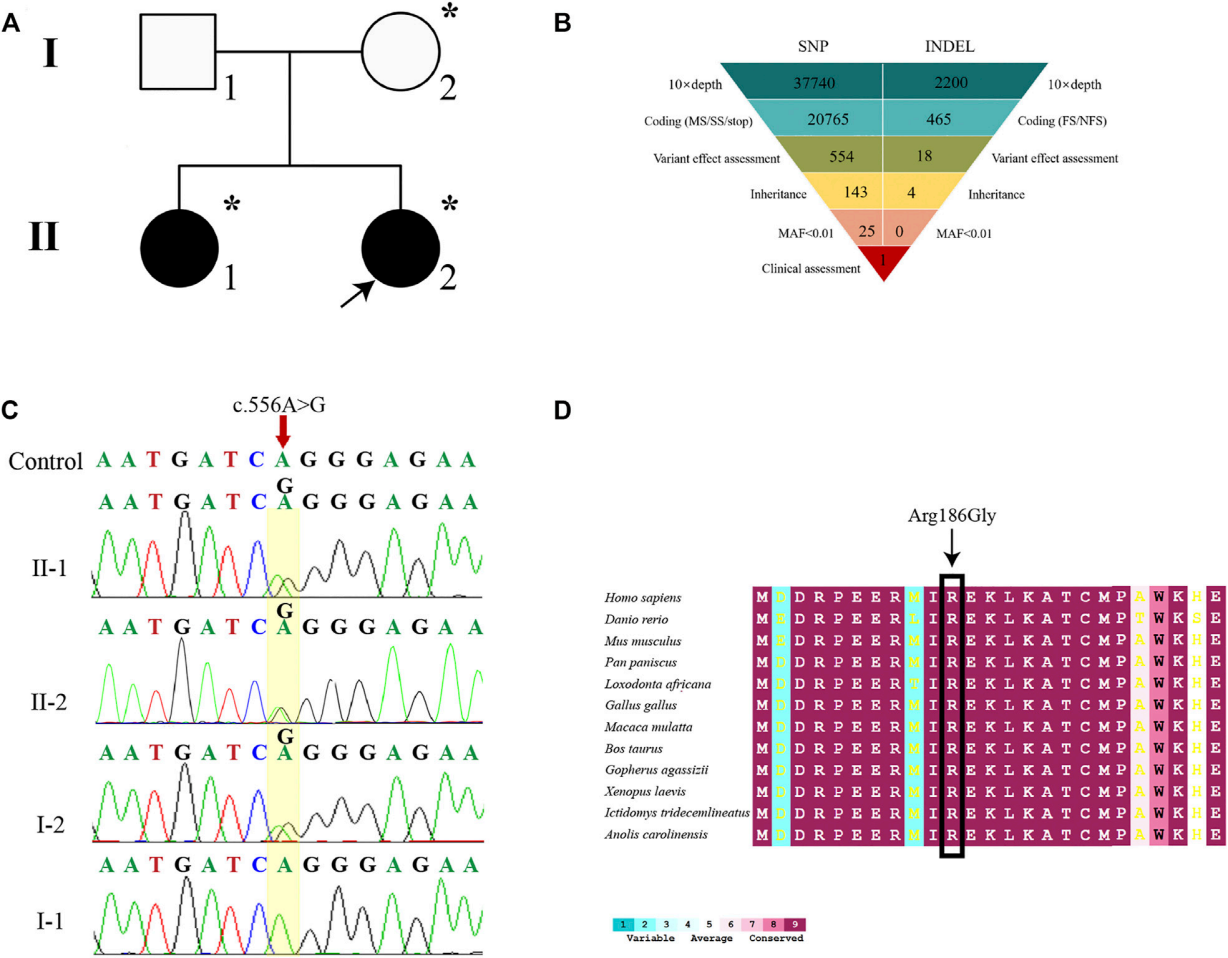
Genetic diagnosis. **(A)** The pedigree with 46, XY DSD. The arrowhead denotes the proband. Squares represent XY individuals, and circles represent XX individuals. Affected individuals are shown as filled with black symbols. Dot-filled symbols represent the unaffected heterozygous carriers. Unfilled symbols indicate clinically unaffected subjects exhibiting the wild-type sequence. **(B)** Schematic representation of the exome-data-filtering approach assumes dominant inheritance in the family. The following abbreviations were used: MS, missense variant; SS, splice-site variant; stop, stop-codon variant; FS, frameshift indel; and NFS, non-frameshift indel. **(C)** Sanger sequencing chromatograms is showing that the proband, her elder sister (II-1), and her mother (I-2) harbor a heterozygous c.556A > G variant of the *MAP3K1* gene. **(D)** Inter-specific conservation analysis of MAP3K1 protein. Color code bars are used to show the conservation of amino acids.

Both WES and Sanger sequencing were performed on samples from the members of a 46,XY DSD core pedigree, including two affected siblings and their parents (Figure 2A). Quality control of FASTQ files was performed, and the proband’s WES satisfied the variant screening criteria (Supplementary Figure S2). There were a total of 21,230 variants in the exome region, out of which 53.15% were nonsynonymous variants (Figures 2B,C). The 11,282 nonsynonymous variants were filtered into 25 rare variants. Finally, a mutation site was identified in the *MAP3K1* gene (c.556A > G) (Figure 2B). The *MAP3K1* gene underwent amplification by PCR. The Sanger sequencing demonstrated that the proband’s mother and elder sister also carried the c.556A > G (r.556a > g, p. R186G) heterozygous variant (Figures 2A,D). Besides, the WES and Sanger sequencing confirmed a missense variant of the *MAP3K1* gene, which is not contained in the public population database gnomAD (http://gnomad), Chinese Millionome database (https://db.cngb.org) or Human Gene Mutation database (http://www.hgmd.org/).

To further analyze the genetic testing data, we assessed the interspecific conservation and pathogenicity of the c.556A > G variant. We compared MAP3K1 protein sequences in 72 vertebrates such as *Homo sapiens*, *Danio rerio*, *Mus musculus*, *Pan paniscus*, *Loxodonta africana*, *Gallus gallus*, *Macaca mulatta*, *Bos taurus*, *Gopherus agassizii*, *Xenopus laevis*, *Ictidomys tridecemlineatus*, and *Anolis carolinensi*, as shown in Figure 2E. ConSurf Server was used for alignment and tint the residue according to the degree of conservation. The residues at position Arg186 of MAP3K1 were highly conserved across all the represented vertebrates (100%) (Figure 2E). The *MAP3K1* missense variant was predicted to be deleterious by all five predictors (PredictSNP, polyphen1, polyphen2, SIFT, and SNAP), with a high confidence score of 0.65, 0.59, 0.60, 0.79, and 0.81, respectively (Supplementary Figure S3). These findings suggested that the c.556A > G (p.R186G) variant in *MAP3K1* gene might be pathogenic.

### Three-Dimensional Structure Modeling

**FIGURE 3:**
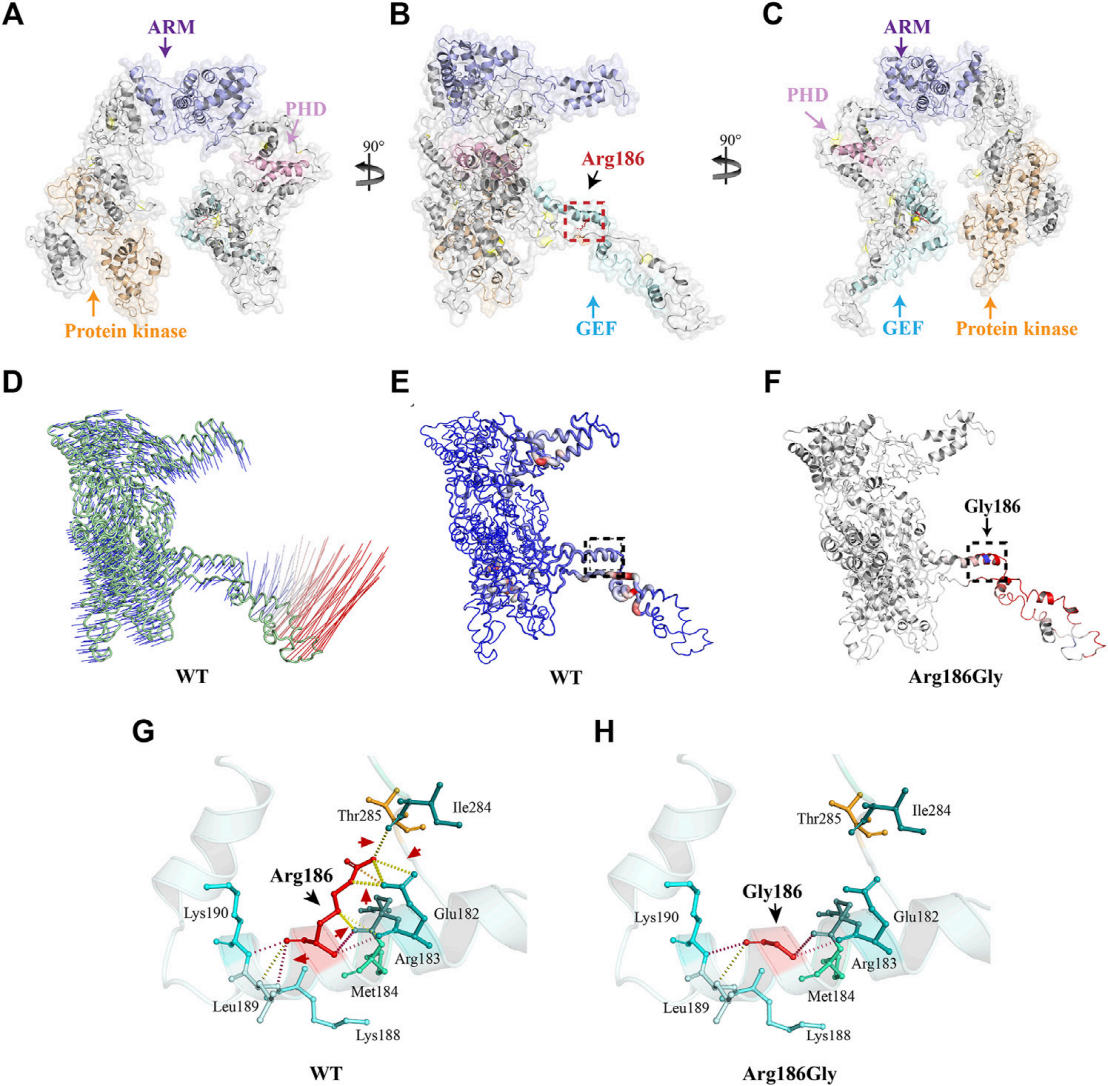
Three-dimensional structure analysis and the protein dynamics of MAP3K1. **(A)** Three-dimensional structure as predicted by the I-TASSER online software. **(B)** As per **(A)**  rotated 90° about the *y*-axis. The black dotted box marks the position of the Arg186 variant. **(C)** As per **(B)**  rotated 90° about the *y*-axis. Yellow indicates the protein kinase domain, purple indicates the ARMadillo repeat (ARM) domain, pink indicates Plant Homeo Domain (PHD), and blue indicates the Guanine Exchange Factor (GEF) domain. The Arg186 amino acid residue of MAP3K1 is depicted as a red stick. **(D)** Vector field representation of molecule for the first non-trivial mode of molecular motion based on Normal Mode Analysis. The short (blue) and long (red) curves represent the change of trajectory. **(E)** Deformation energy of wild-type MAP3K1: The magnitude of the deformation/fluctuation is represented by thin to thick tubes colored blue (low), white (moderate), or red (high). The black dotted box marks represent the position of Arg186. **(F)** Δ Vibrational Entropy Energy | Visual representation of variant: Amino acids colored based on the vibrational entropy change of the variant. Blue represents a rigidification of structure. Red represents a gain in flexibility. **(G,H)** Interaction prediction between amino acid residues: Wild-type and variant residues are represented as red sticks. These are placed alongside surrounding residues which are also involved in other types of interactions. Red arrows indicate interactions between altered amino acids. Black arrows indicate wild-type or variant amino acid residues. Hydrogen bonding is shown with a yellow dotted line.

To further understand the structural changes in the MAP3K1 protein, we used the I-TASSER online software to perform *ab initio* modeling of the MAP3K1 structure (Figures 3A–C). C-score, a confidence rating factor with values in [−5,2], was used to evaluate the quality of the I-TASSER predictions. Our analysis showed that the MAP3K1 model obtained by the I-TASSER server had a high C-score (0.56–0.80) and TM-score, with a structural similarity measurement with values in [0,1], correlation coefficient (0.91), thus demonstrating that the predictions were credible. The p. R186G variant was located at the middle of the GEF domain (amino acids 164–231) and in the predicted alpha-helix of the MAP3K1 protein.

The I-TASSER prediction results were then used as a basis for further modelling the protein structure to predict possible changes in protein flexibility after mutation. The DynaMut web server was used to analyze and visualize protein dynamics, and assess the impact of mutations on the protein dynamics. We showed a molecular vector field representation of the first non-trivial mode of the molecule motion based on Normal Mode Analysis, as shown in Figure 3D. Figure 3E shows deformation energy performed over the first ten non-trivial modes of the molecule. This data provides a measure for local flexibility in the wild-type MAP3K1 protein. The data showed that the flexibility of the GEF domain is highest of all domains within the MAP3K1 protein. In addition, the p. R186G variant further increased the flexibility of the GEF domain (ΔΔSVibENCoM: 0.828 kcal/mol/K) (Figure 3F).

Finally, we predicted the local interactions between the wild-type and variant amino acid residues via the DynaMut online server. The data demonstrated that the variant led to a change from arginine (Arg) to glycine (Gly), plus significant charge differences. In particular, the loss of charge of the arginine residue could potentially result in other interaction losses with other molecules or residues. In addition, there was introduction of a more hydrophobic residue into the variant, which could trigger loss of hydrogen bonds and/or disruption of protein folding (Figures 3F,G). Thus, the unique structural disturbance of the p. R186G variant altered the local hydrogen bond pattern of the MAP3K1 protein, which might be responsible for the increased flexibility of the variant.

### Changed Interaction Between MAP3K1^R186G^ and RhoA or Ub

**FIGURE 4:**
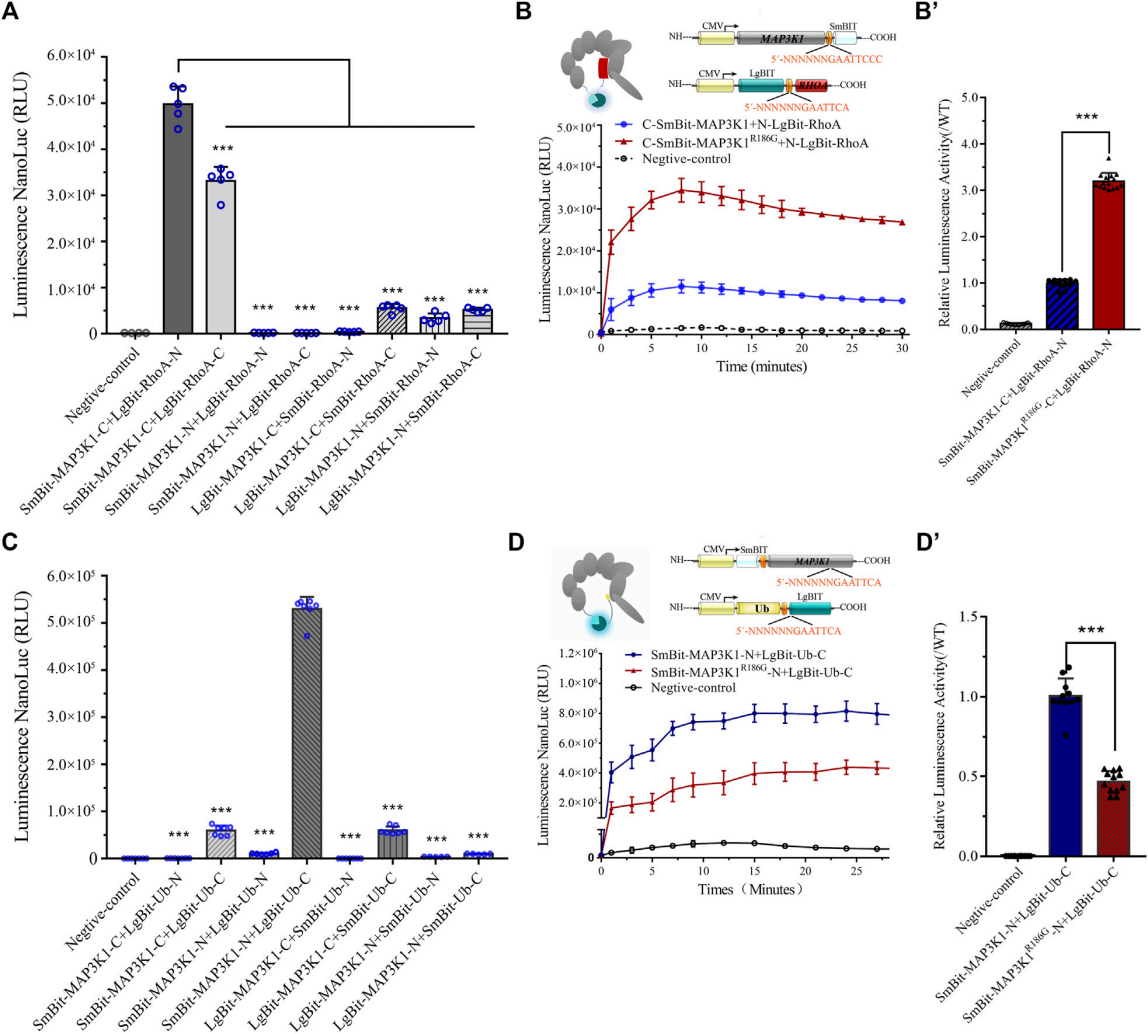
Changed interaction between MAP3K1 and RhoA or Ub. **(A,B)** The capacity of wild-type (MAP3K1) and variant (MAP3K1^R186G^) of MAP3K1 to bind RhoA. **(A)** LgBiT and SmBiT fusion expression vectors with *MAP3K1* and *RHOA* gene were established and instantaneously transfected into HEK293T cells with 8 different combinations. The combination of maximum response signals was filtered. Each dot in the graphs represents a single repetition (*n* = 5; mean ± SEM shown; ***p < 0.001 by *t*-test). **(B)** Schematic structure of MAP3K1 and RhoA NanoBiT-fused proteins. The *MAP3K1* gene and the C-terminus of SmBiT were fused to form the *MAP3K1*-SmBiT-C plasmid. The *RHOA* gene and the N-terminus of LgBiT were fused to form the *RHOA*-LgBiT-N expression plasmid. Negative control was transfected with only the *MAP3K1*-SmBiT-C plasmid. Twenty-4 hours after co-transfection, the diluted substrate was directly added to each well to measure the absolute value of NanoBiT activity for 30 min. **(B)** For comparison, the mean wild-type values at each time point were adjusted to 1′, and each black triangle represents the relative value of MAP3K1-RhoA binding capacity in the mutant group at the corresponding time point. HEK293T cells transfected with only Smbit-MAP3K1-C plasmid used as a negative control. **(C,D)** The ability of wild-type (MAP3K1) and variant (MAP3K1^R186G^) of MAP3K1 to bind Ub. **(C)** LgBiT and SmBiT fusion expression vectors with *MAP3K1* and *UB* genes were determined and instantaneously transfected into HEK293T cells with 8 different combinations. Each dot in the graphs represents a single repetition. **(D)** Schematic structure of the MAP3K1 and Ub NanoBiT-fused proteins. The *MAP3K1* gene and the N-terminus of SmBiT were fused to form the SmBiT-*MAP3K1*-N plasmid. The *UB* gene and the C-terminus of the LgBiT were fused to form the *UB*-LgBiT-C expression plasmid. Negative control was transfected with the SmBiT-*MAP3K1*-N plasmid only. Twenty-4 hours after co-transfection the absolute value of NanoBiT activity for 30 min was measured (*n* = 7; mean ± SEM shown; ***p < 0.001 by *t*-test). **(D′)** The mean wild-type values at each time point were adjusted to 1′, and each black triangle represents the relative value of MAP3K1-Ub binding capacity in the mutant group at the corresponding time point. HEK293T cells transfected with only Smbit-MAP3K1-N plasmid were used as a negative control. HEK293T cells transfected with only Smbit-MAP3K1-N plasmid were used as a negative control. Each value represents the mean ± SD from at least three independent cultures (***p < 0.001 by *t*-test).

To examine if the MAP3K1^R186G^ variant altered its interaction with other cofactors, we used a luciferase-based protein fragment complementation assay (NanoBiT), to measure its ability to bind RhoA or Ub. Co-expression of the SmBit-MAP3K1-C and LgBit-RhoA-N yielded a clear luminescent signal. This finding suggested a strong interaction for the receptors in this combination (Figure 4A). As shown in Figures 4B,B', the binding of the variant to RhoA was significantly increased to 3.19 ± 0.18 fold compared to the wild-type protein. The interaction between MAP3K1 and Ub was simultaneously analyzed using the same method (Figures 4C,D). The binding ability of this variant to Ub was reduced to 43–49% of that of the wild-type (Figures 4D,D'). These data indicated that MAP3K1^R186G^ had increased binding affinity towards RhoA but reduced affinity for Ub.

**FIGURE 5:**
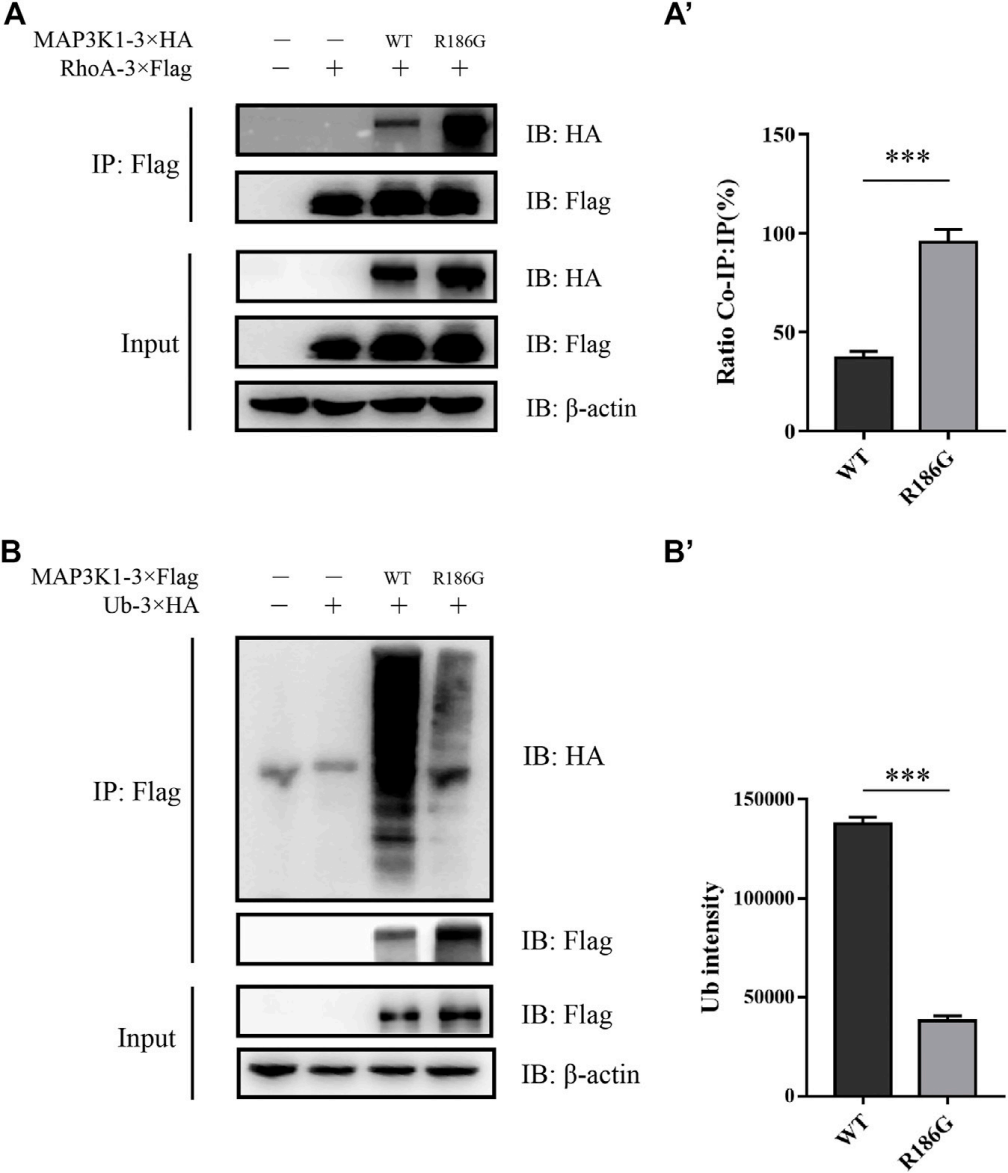
Co-IP assay analysis of the interaction between MAP3K1 and RhoA or Ub. **(A)** Co-IP assay analysis of the interaction between MAP3K1 wild-type and R186G variant with RhoA. Cells were co-transfected with MAP3K1-3×HA and RHOA-3×Flag in HEK293T cells; Some of the lysates were subjected to input assays to assess β-actin, MAP3K1-HA, and RhoA-Flag protein levels, and the remainder were subjected to IP assays. **(A′)** Quantification of Co-IP western blots. Band intensities were determined and used to calculate the relative ratio of Co-IP to IP signal. Data shown are averaged percentages ±SEM (*n* = 3 independent experiments, ***p < 0.001 by Mann-Whitney *U* test). **(B)** The ubiquitination of MAP3K1 wild-type and R186G variants in HEK293T cells was Co-IP then immunoblotted. **(B′)** Quantification of MAP3K1 polyubiquitination by ImageJ. Data shown are Mean ± SEM (*n* = 3 independent experiments, ***p < 0.001 by Mann-Whitney *U* test).

RhoA and Ub have a common binding motif. RhoA interacts with the GEF and PHD domains (residues 149–636) of MAP3K1 (Gallagher et al., 2004). On the other hand, Ub interacts with the PHD domains (residues 438–440) of MAP3K1 (Charlaftis et al., 2014). The variant site (p.R186G) is located in the RhoA and MAP3K1 binding regions. A previous study showed that a combination of MAP3K1 and RhoA may inhibit MAP3K1-specific ubiquitination of other substrates, including auto-ubiquitination (Gallagher et al., 2004). Our Co-IP assay confirmed that the binding capacity of MAP3K1^R186G^ to RhoA was 2.57 times that of the wild-type protein (Figures 5A,A'), and the ubiquitination degree of the wild-type MAP3K1 protein was 3.6-fold that of the MAP3K1^R186G^ variant (Figures 5B,B').

### Hyperactivation of Wnt4/β-Catenin Signaling Pathway

**FIGURE 6:**
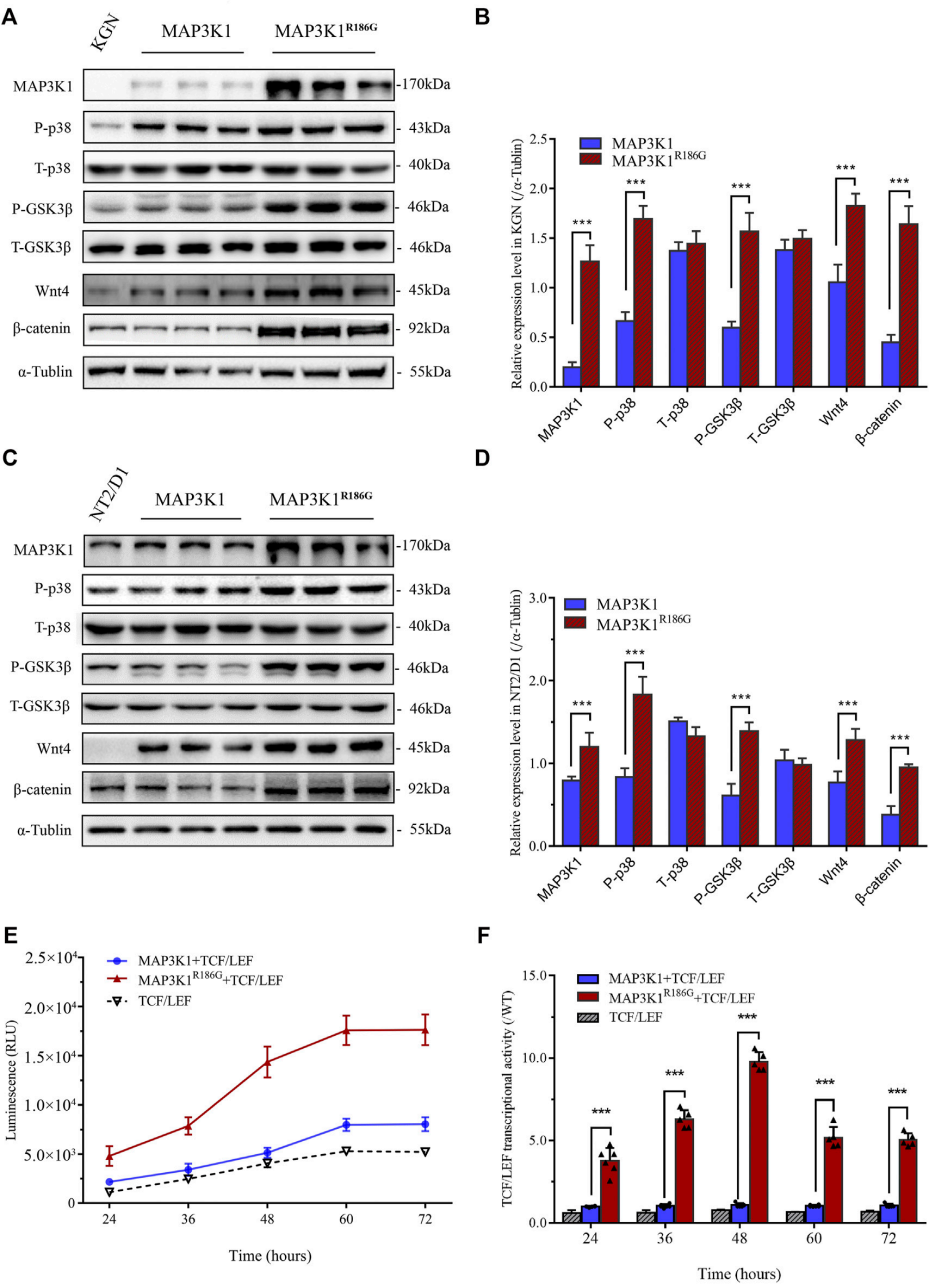
Hyperactivation of Wnt4/β-catenin signaling. **(A–D)** NT2D1 or KGN cells transfected with empty vector plasmid or wild-type (MAP3K1) or mutant MAP3K1 (MAP3K1^R186G^) were subjected to Western blotting analysis. **(A)** Western blotting analysis of phosphorylated P38 (P-p38), total p38 (T-p38), phosphorylated GSK3β (P-GSK3β), total GSK3β (T-GSK3β), Wnt4, and β-catenin in KGN cells. *n* = 3; mean ± SEM shown; ***p < 0.001 by *t*-test. **(B)** Quantified analysis of Western blotting of P-p38, T-p38, P-GSK3β, T-GSK3β, Wnt4, and β-catenin in KGN cells. **(C)** Western blotting analysis of P-p38, T-p38, P-GSK3β, T-GSK3β, Wnt4, and β-catenin in NT2/D1 cells. *n* = 3; mean ± SEM shown; ***p < 0.001 by *t*-test. **(D)** Quantified Western blotting analysis of P-p38, T-p38, P-GSK3β, T-GSK3β, Wnt4, and β-catenin in NT2/D1 cells. The data were expressed as the ratio of proteins level (gray value) to α-Tubulin protein level (gray value). **(E,F)** Co-transfected 293T cells with wild-type or mutant MAP3K1 expression plasmids and TCF/LEF-TOP flash reporter plasmid. **(E)** luciferase activity was determined at 24 h, 36 h, 48 h, 60 h, and 72 h after transfection. **(F)** For comparison, the wild-type values were adjusted to 1’; a histogram represents the relative value of transcriptional activities. Dots represent the mean of each experiment. *n* > 3; mean ± SEM shown; ***p < 0.001 by *t*-test.

RhoA is a potent activator of MAP3K1 through activation of its kinase (Gallagher et al., 2004). Glycogen synthase kinase 3β (GSK3β) is a classic negative regulator of Wnt/β-catenin signaling pathway and phosphorylation of GSK3β results in its inactivation. p38 regulates canonical Wnt/β-catenin signaling by inactivation of the GSK3β (Bikkavilli et al., 2008). To determine the effect of the MAP3K1^R186G^ variant on kinase and ovarian-specific pathways, we evaluated phosphorylation levels of p38 and GSK3β as well as activation of the Wnt4/β-catenin pathway in the testicular teratoma cells (NT2/D1) and ovary-derived granular cells (KGN). The NT2/D1 and KGN cells were transiently transfected with expression plasmids, containing either the wild-type or mutant *MAP3K1* gene. The MAP3K1 protein levels were significantly elevated in the mutant group compared to the wild-type group. The protein expression of the MAP3K1^R186G^ variant was 6.4 and 2.0 times higher than that of the wild type in the KGN and NT2/D1 cells, respectively (Figures 6A–D). In the KGN ovarian cells, the phosphorylated p38 (P-p38) and GSK3β (P-GSK3β) protein levels were 2.5 and 2.6-fold higher, respectively, in the mutant group compared to those in the wild-type group. Moreover, our results demonstrated that the variant could activate the Wnt4/β-catenin signaling. The mutant group showed 1.7 and 3.7-fold increase in the Wnt4 and β-catenin protein levels, respectively, compared to the wild-type group in the KGN cells (Figures 6A,B). Similarly, in the NT2/D1 testicular cells, phosphorylated p38 and GSK3β proteins were significantly elevated in the mutant group, which were 2.3 and 2.2 times higher than those in the wild-type group, respectively. Wnt4 and β-catenin levels had also increased to about 1.7 and 2.5-fold in the mutant group compared with the wild-type group (Figures 6C,D). Furthermore, we analyzed the transcriptional activity of the variant on β-catenin using a TCF/LEF luciferase assay. The data showed that at 48 h after transfection, the relative activity of TCF/LEF in the mutant group reached its peak, which was 9.78 ± 0.57 times that of the wild-type group (Figures 6E,F). These results suggested that the MAP3K1^R186G^ variant enhances the phosphorylation of p38 and GSK3β, leading to the accumulation of β-catenin and activation of the Wnt4/β-catenin signaling pathway.

### Increased Expression of Pro-ovarian Transcription Factor FOXL2

**FIGURE 7:**
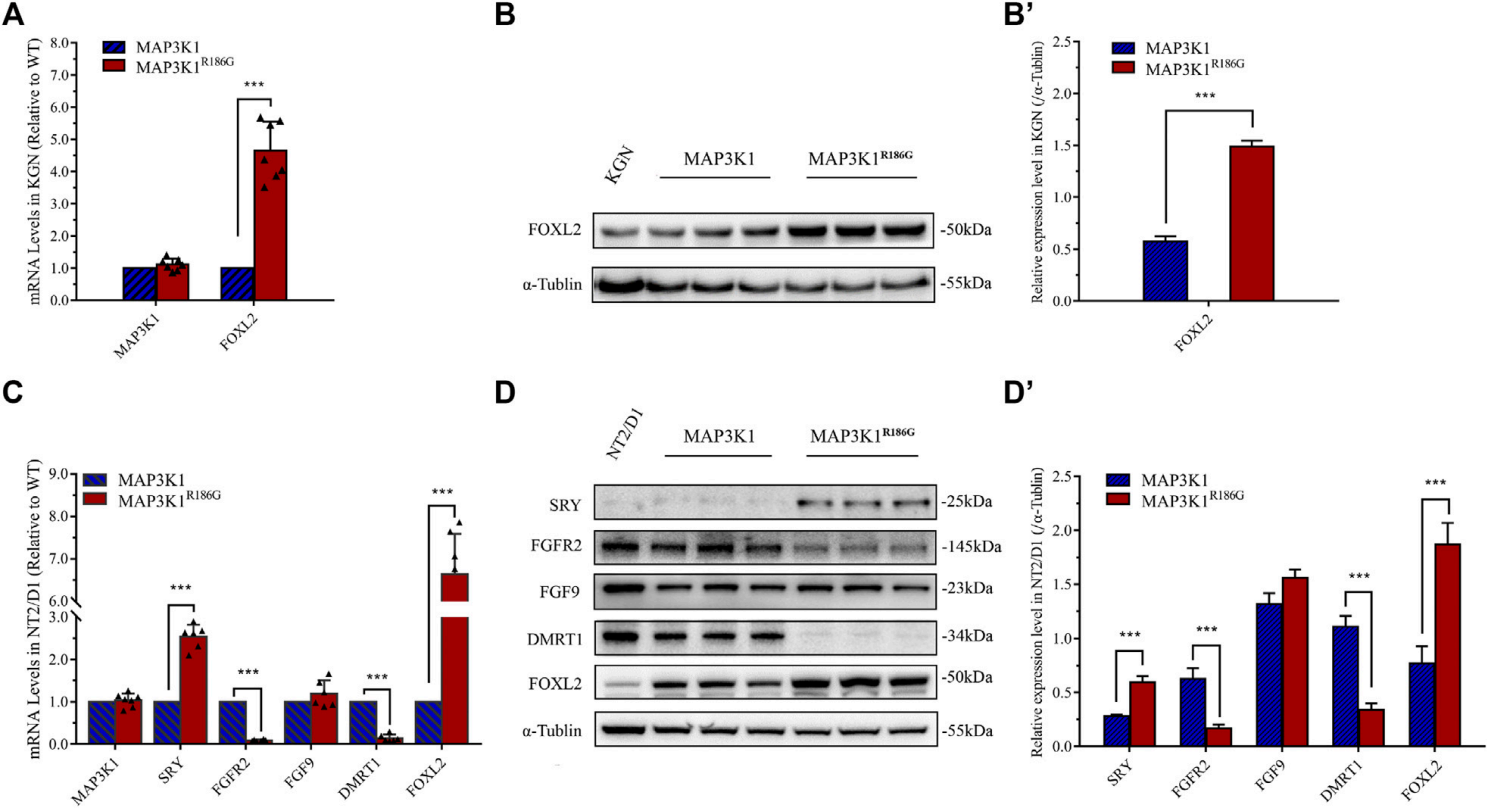
Upregulated expression of pro-ovarian transcription factor *FOXL2* gene. **(A,B)** KGN cells were transfected with empty vector plasmids expressing either wild-type (MAP3K1) or mutant (MAP3K1^R186G^) MAP3K1 genes. **(A)** mRNA levels of MAP3K1 and FOXL2 were measured using quantitative PCR. **(B)** Western blotting analysis of MAP3K1 and FOXL2. **(B′)** Quantified analysis of western blotting of FOXL2. **(C,D)** NT2/D1cells were transfected with empty vector plasmids expressing either wild-type (MAP3K1) or variant (MAP3K1^R186G^) of the MAP3K1 gene. **(C)** mRNA levels of MAP3K1, SRY, FGFR2, FGF9, DMRT1, and FOXL2 were measured using quantitative PCR. **(D)** Western blotting analysis of SRY, FGFR-2, FGF-9, DMRT1, and FOXL2. **(D′)** Quantified analysis of Western blotting of MAP3K1, SRY, FGFR-2, FGF-9, DMRT1, and FOXL2. For quantitative PCR, results were normalized to β-actin as a reference and compared with control cells transfected with an empty vector plasmid (*n* ≥ 5; mean ± SEM shown; ***p < 0.001 by Student t-test). For Western blotting, results were normalized to α-tubulin as a reference and compared with control cells transfected with empty plasmids (*n* = 3; mean ± SEM shown; *p < 0.05, **p < 0.01 and ***p < 0.001).

To further assess the effect of the MAP3K1^R186G^ variant on sex-specific signaling pathways, we analyzed the effects of the variant on the expression of critical genes in testis and ovarian differentiation. Forkhead box L2 (*FOXL2*), a pro-ovarian transcription factor, is essential for ovarian differentiation and functions (Elzaiat et al., 2017; Nicol et al., 2019). Fibroblast growth factor 9 (FGF-9) and its receptor, fibroblast growth factor receptors 2 (FGFR-2), as well as doublesex and mad-3 related transcription factor 1 (DMRT1), are critical regulators of testicular determination and mediate inhibition of ovarian pathways (Bagheri-Fam et al., 2017; Huang et al., 2017; Harpelunde Poulsen et al., 2019). We overexpressed the wild-type or mutant *MAP3K1* gene in the KGN ovarian granulosa cell line. Our analysis showed that there was no significant difference in *MAP3K1* mRNA expression between the wild-type and mutant groups (Figures 7A,C). Furthermore, the FOXL2 mRNA expression in the mutant group was 4.1-fold that in the wild-type group (Figure 7A), while the FOXL2 protein levels were 2.6-fold that in the wild-type group (Figures 7B,B'). We then assessed the mRNA and protein levels of *SRY*, *FGFR2*, *FGF9*, *DMRT1*, and *FOXL2* genes in the NT2/D1 testicular teratoma cells transfected with the mutant or wild-type MAK3K1 expression plasmid, respectively. The expression of *FGFR2* mRNA in the mutant group was 8.5% of that in the wild-type group (Figure 7C), while the protein expression had decreased to 26.5% of that in the wild-type (Figures 7D,D'). The mRNA expression of *DMRT1* in the mutant group was 14.1% of that in the wild-type group (Figure 7C), and the protein expression had decreased to 30.5% of the wild-type (Figures 7D,D'). The mRNA and protein expression of *FOXL2* gene was 4.1 and 2.4-fold higher, respectively, in the mutant group compared to that in the wild-type group (Figures 7C,D,D'). These results demonstrated that the MAP3K1^R186G^ variant inhibited the expression of the *DMRT1* gene in the testis cells but promoted the expression of the *FOXL2* gene in both the testis and ovarian cells. Unexpectedly, the mRNA and protein levels of SRY were 2.6-fold and 2.4-fold higher, respectively, in the mutant group compared to the control group.

## Discussion

This work describes two patients in a Chinese family with clinical features of 46, XY DSD. Diagnosis at the time of birth has proved to be difficult since the external genitalia of these patients harbors a feminine appearance. Due to the undeveloped secondary sexual characteristics and/or germ cell tumors, treatment is often sought during adolescence. Unlike the general population, such patients have a significant risk of developing germ cell neoplasms, specifically gonadoblastoma and dysgerminoma (Cools et al., 2006). Using WES and Sanger sequencing, a heterozygous missense variant of the *MAP3K1* gene, c.556A > G (p.R186G) was discovered from a Chinese family, including a healthy mother (46, XX) and her two daughters with 46, XY DSD. MAP3K1 protein comprises five domains, including the Guanine Exchange Factor (GEF) domain, SWI2/SNF2, and MuDR (SWIM) domain, the Plant Homeo Domain (PHD), the ARMadillo repeat (ARM) domain, and the Protein kinase domain (Uhlik et al., 2004; Chamberlin et al., 2019). So far, at least 21 different deleterious MAP3K1 gene variants have been identified, resulting in 46,XY DSD; out of these, 18 are missense variants, two are splice site variants, and one is a small deletion variant (Kim et al., 2017; Chamberlin et al., 2019; Xue et al., 2019). These variants are concentrated in the GEF and ARM domains of MAP3K1. Arg186 of MAP3K1 is located in the α-helix of the GEF domain; it is highly and evolutionarily conserved in vertebrates. Several pathogenicity prediction servers predict that the p. R186G variant would be deleterious. We used I-TASSER to predict its three-dimensional structure and to further understand the structural changes of the variant. Also, the prediction results of the DynaMut online server showed that the local structure of the p. R186G variant had significantly changed, and the flexibility of the local protein increased with unique structural disturbance of MAP3K1^R186G^ including changes in local hydrogen bond patterns for MAP3K1. Known protein-protein interactions, protein-substrate binding, or protein conformation transformation require the flexibility of an α-helix to guarantee the protein function and activity (Jarosch, 2005; Pauwels and Tompa, 2016). This could be the reason for the functional modification of this variant.

**FIGURE 8:**
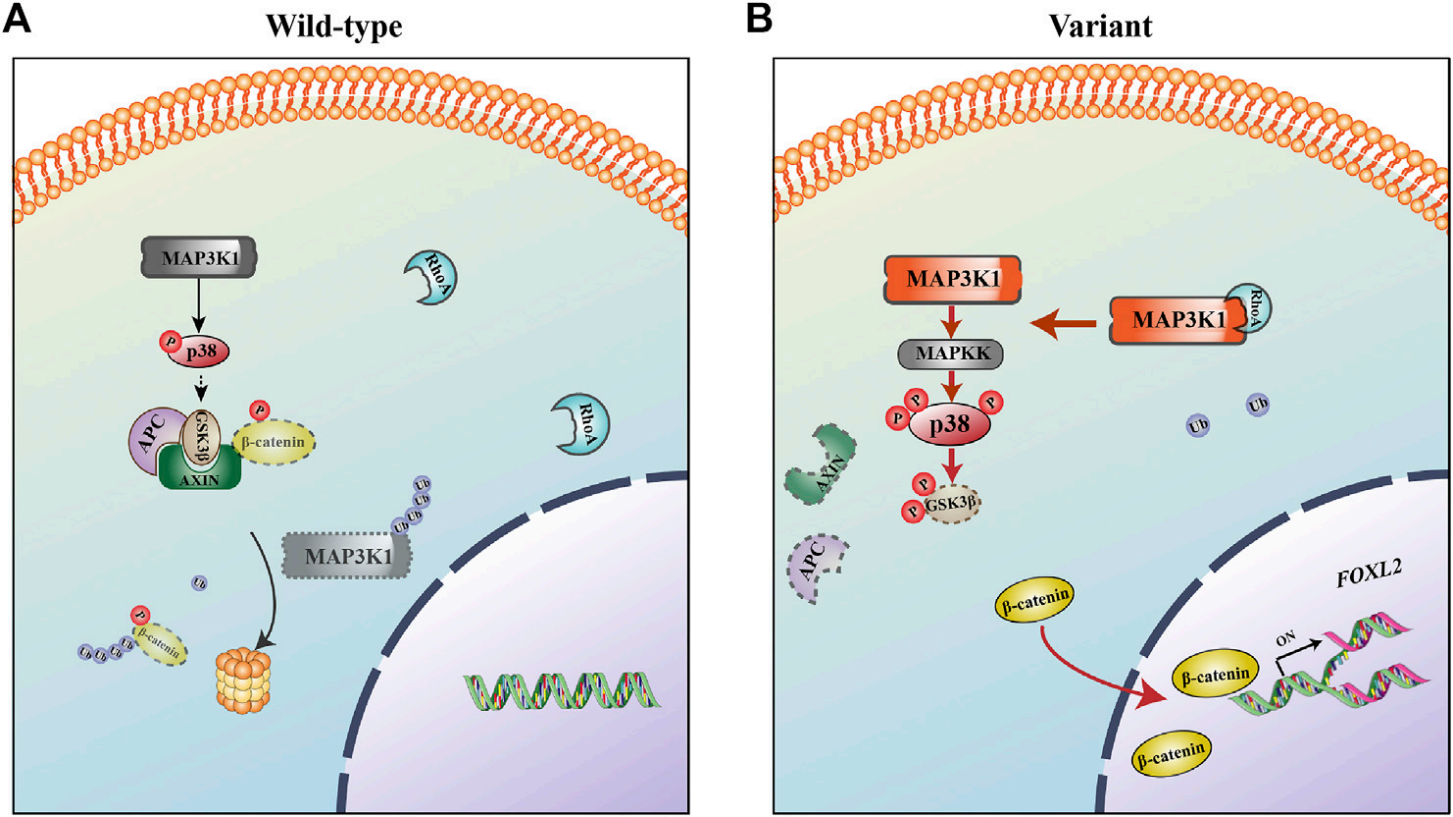
The mechanism for 46, XY DSD as caused by the MAP3K1^R186G^ variant. **(A)** In the wild-type, MAP3K1 tends to be degraded by auto-ubiquitination. **(B)** In the mutant, MAP3K1^R186G^ enhances its binding to RhoA and stability, causing hyperphosphorylation of p38 and GSK3β, activating the Wnt4/β-catenin/FOXL2 pathway, and in turn resulting in 46, XY DSD.

The MAP3K1^R186G^ protein level was significantly higher than those of the wild-type, whereas its mRNA level was similar. MAP3K1 is the only mitogen-activated protein kinase (MAPK) family member with both protein kinase and E3 ubiquitin ligase activities (Gallagher et al., 2004). RhoA is a strong activator of MAP3K1, promoting MAP3K1 kinase activity by up to 10 times its former level (Gallagher et al., 2004). The effects of MAP3K1^R186G^ were evaluated on the binding of cofactor RhoA. Both protein complementation and Co-IP experiments reveal that the binding capacity of MAP3K1^R186G^ to RhoA was also significantly increased. On the other hand, RhoA competitively inhibits its capacity to ubiquitinate other proteins, including itself via its binding to MAP3K1 (Gallagher et al., 2004; Charlaftis et al., 2014). In this way, we noted a reduced binding of the MAP3K1^R186G^ variant to Ub. The ubiquitination level of MAP3K1^R186G^ was lower than that of the wild-type, thereby suggesting increased stability of this variant. This could explain why MAP3K1^R186G^ protein is overexpressed in NT2/D1 cells, and why silencing RhoA with siRNA can phenotypically restore NT2/D1 cells with *MAP3K1* variant overexpression (Loke et al., 2014). Therefore, the MAP3K1^R186G^ variant increased its binding with RhoA, thus competitively inhibiting its binding force with Ub, which could then be responsible for increased MAP3K1^R186G^ protein levels. Summarily, MAP3K1^R186G^ enhances its binding to RhoA and stability, causing hyperphosphorylation of p38 and GSK3β, in turn activating the Wnt4/β-catenin pathway (Figure 8).

The process of human gonadal development is highly complex and accompanied by the expression of several genes (Baetens et al., 2019; Wisniewski et al., 2019). In the 6th week of human embryonic life, *SRY* upregulation (OMIM *480,000) in XY individuals causes the development of primitive gonadal glands into a testis. In XX individuals, bipotential gonads differentiate as ovaries, as driven by *WNT4* (OMIM*603,490) and *CTNNB1* (β-catenin) (OMIM*116,806). In the human embryonic life stage, many sex-determining regulators, including *FGF9* (OMIM*600,921), *FGFR2* (OMIM*176,943), *DMRT1* (OMIM*602,424), and *FOXL2* (OMIM*605,597) (Ottolenghi et al., 2007; Bikkavilli et al., 2008; Bagheri-Fam et al., 2017; Huang et al., 2017) are involved in strict regulation of development and maintenance of testicular or ovarian pathways (Chassot et al., 2008; Eggers et al., 2014). Of note, sex differentiation of bipotential gonads is a vital step in determining the reproductive identity of an embryo. This cell fate specification is based on an accurate balance of transcriptional control. In our study, the MAP3K1^R186G^ variant upregulated the expression of genes associated with ovarian development (including *WNT4*, *CTNNB1*, and *FOXL2*) and downregulated the expression of testicular development-related genes (*FGFR2* and *DMRT1*) in NT2/D1 cells. Nevertheless, it remains unclear which specific signaling pathway plays the most predominant or initiating role in 46, XY DSD caused by *MAP3K1* gene variants (Pearlman et al., 2010). For further validation, we applied an ovarian-derived KGN cell line lacking the SRY signaling pathway. Corresponding to the results obtained in NT2-D1 cells, hyperactivation of the Wnt4/β-catenin-FOXL2 pathway was detected in KGN cells. MAPK-p38 regulates canonical Wnt/β-catenin signaling by inactivating GSK3β by *Thr* (390) phosphorylation (Bikkavilli et al., 2008; Loke et al., 2014; Thornton et al., 2016). This study demonstrates that the MAP3K1^R186G^ variant triggers abnormal hyperphosphorylation of p38 and GSK3β in cells. Phosphorylated p38 promotes the phosphorylation of GSK3β*,* weakening its activity and in turn causing the stabilization of β-catenin and increased transcriptional activity. Herein, *SRY* gene expression in the mutant group was upregulated, which may be linked to hyperphosphorylation of p38. Nevertheless, as previously suggested, *SRY* only acts as an “on/off”; it is instantaneously expressed within a concise time window and when it reaches its threshold, it activates downstream pathways and promotes testicular formation (Sekido and Lovell-Badge, 2009). Therefore, the activation of downstream pathways is critical to the normal development of the testis. *FOXL2* overactivation stimulates Sertoli cells into a granulosa-like cell transformation, even after sex determination (Georges et al., 2013; Li et al., 2017). Notably, *DMRT1* is involved in fetal testicular development and adult testicular maintenance. *DMRT1* gene deletion or inactivation in humans induces XY male-to-female sex reversal (Murphy et al., 2015; Huang et al., 2017). Our results demonstrate that the *MAP3K1* variant upregulates *FOXL2* gene expression and downregulates the expression of *DMRT1*.

In conclusion, we have described the clinical characteristics of two siblings with 46, XY DSD attributed to a missense variant of *MAP3K1* (c.556A > G/p.R186G). As a consequence, MAP3K1^R186G^ presents a higher affinity binding to RhoA and a lower affinity to Ub. This subsequently enhances p38 phosphorylation and glycogen synthase kinase 3β (GSK3β), promotes hyperactivation of Wnt4/β-catenin-FOXL2 signaling, and 46, XY DSD. Our findings provide novel insights into the clinical evaluations and molecular basis of 46, XY DSD.

## Data Availability

The datasets presented in this study can be found in online repositories. The names of the repository/repositories and accession number(s) can be found in the article/Supplementary Material.
