# Supplementary material for: MAP3K1 Variant Causes Hyperactivation of Wnt4/β-Catenin/FOXL2 Signaling Contributing to 46,XY Disorders/Differences of Sex Development
Source: Front Genet. 2022 Mar 3;13:736988. doi: 10.3389/fgene.2022.736988 (PMC8927045; doi:10.3389/fgene.2022.736988)
Supplement: Supplementary file 1 [file Table1.DOCX]

**Table S1. Primer list for qPCR**

| Gene | Gene ID | NCBI Reference Sequence | Forward primers  5’→3’ | Reverse primers  5’→3’ |
| --- | --- | --- | --- | --- |
| MAP3K1 | 4214 | NM_005921 | CCAGACCAGTATCTCAGGAGATG | CCGCTAAACTGTGGCAAGGAGT |
| SRY | 6736 | NM_003140 | GGATGACTGTACGAAAGCCACAC | TTTGTCCAGTGGCTGTAGCGGT |
| SOX9 | 6662 | NM_000346 | AGGAAGCTCGCGGACCAGTAC | GGTGGTCCTTCTTGTGCTGCAC |
| FGFR2 | 2263 | NM_022970 | GTGCCGAATGAAGAACACGACC | GGCGTGTTGTTATCCTCACCAG |
| FGF9 | 2254 | NM_002010 | CCAGGAAAGACCACAGCCGATT | CCATACAGCTCCCCCTTCTCAT |
| CTNNB1 | 1499 | NM_001098209 | CACAAGCAGAGTGCTGAAGGTG | GATTCCTGAGAGTCCAAAGACAG |
| WNT4 | 54361 | NM_03C0761 | GCTGGAGAAGTGCGGCTGTGA | CCACAAACGACTGTGAGAAGGC |
| FOXL2 | 668 | NM_023067 | CGGAGAAGAGGCTCACGCTGT | CTGAGGTTGTGGCGGATGCTAT |
| DMRT1 | 1761 | NM_021951 | CCTTATGTGCCTGGTCAGACAG | GCATCCTCAAAAGAGAAAAACTGG |
| ACTB | 60 | NM_001101 | CACCATTGGCAATGAGCGGTTC | AGGTCTTTGCGGATGTCCACGT |
