# Supplementary figures and images for: MAP3K1 Variant Causes Hyperactivation of Wnt4/β-Catenin/FOXL2 Signaling Contributing to 46,XY Disorders/Differences of Sex Development
Source: Front Genet. 2022 Mar 3;13:736988. doi: 10.3389/fgene.2022.736988 (PMC8927045; doi:10.3389/fgene.2022.736988)

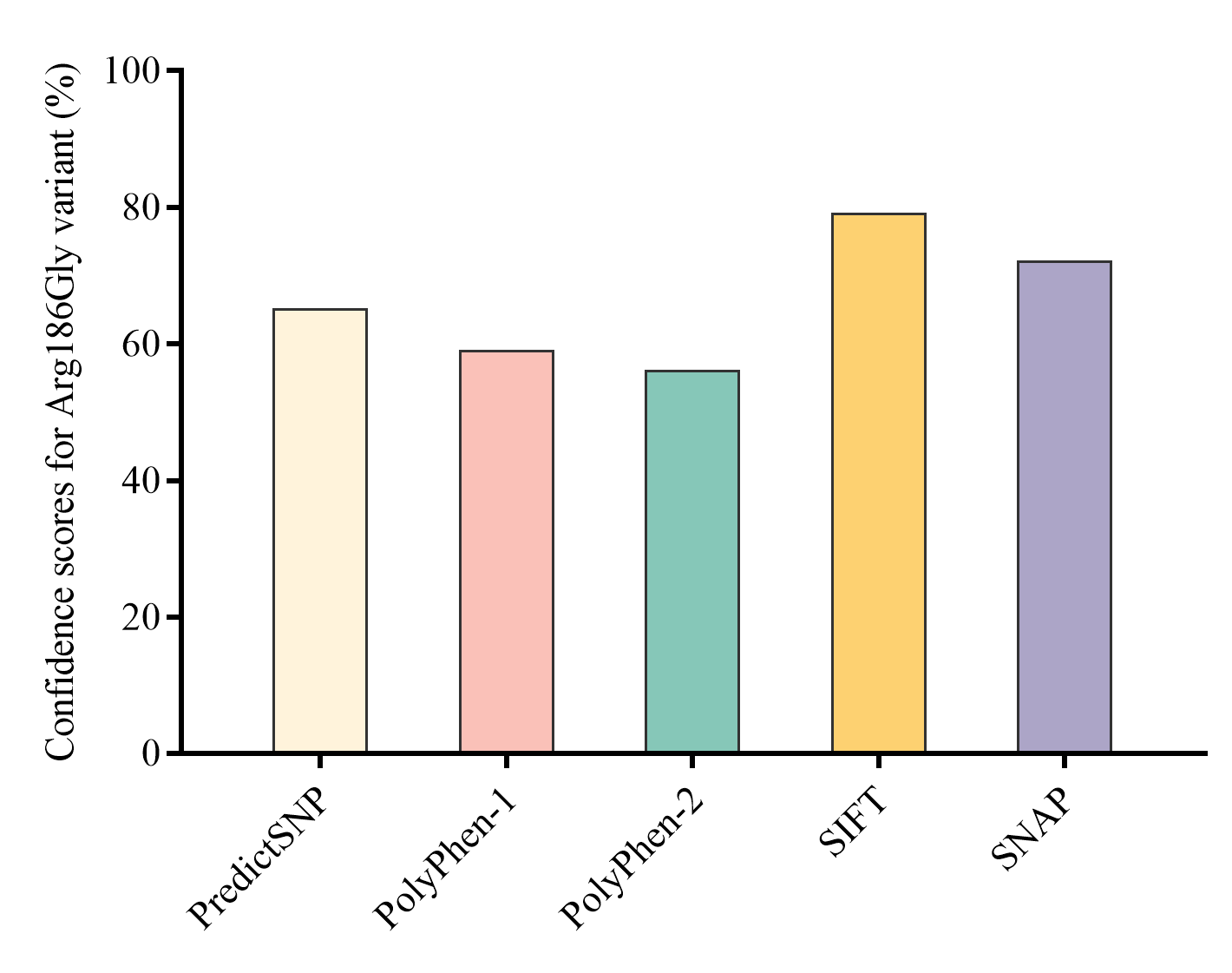

Supplement: Supplementary file 2 [file Image3.TIF]

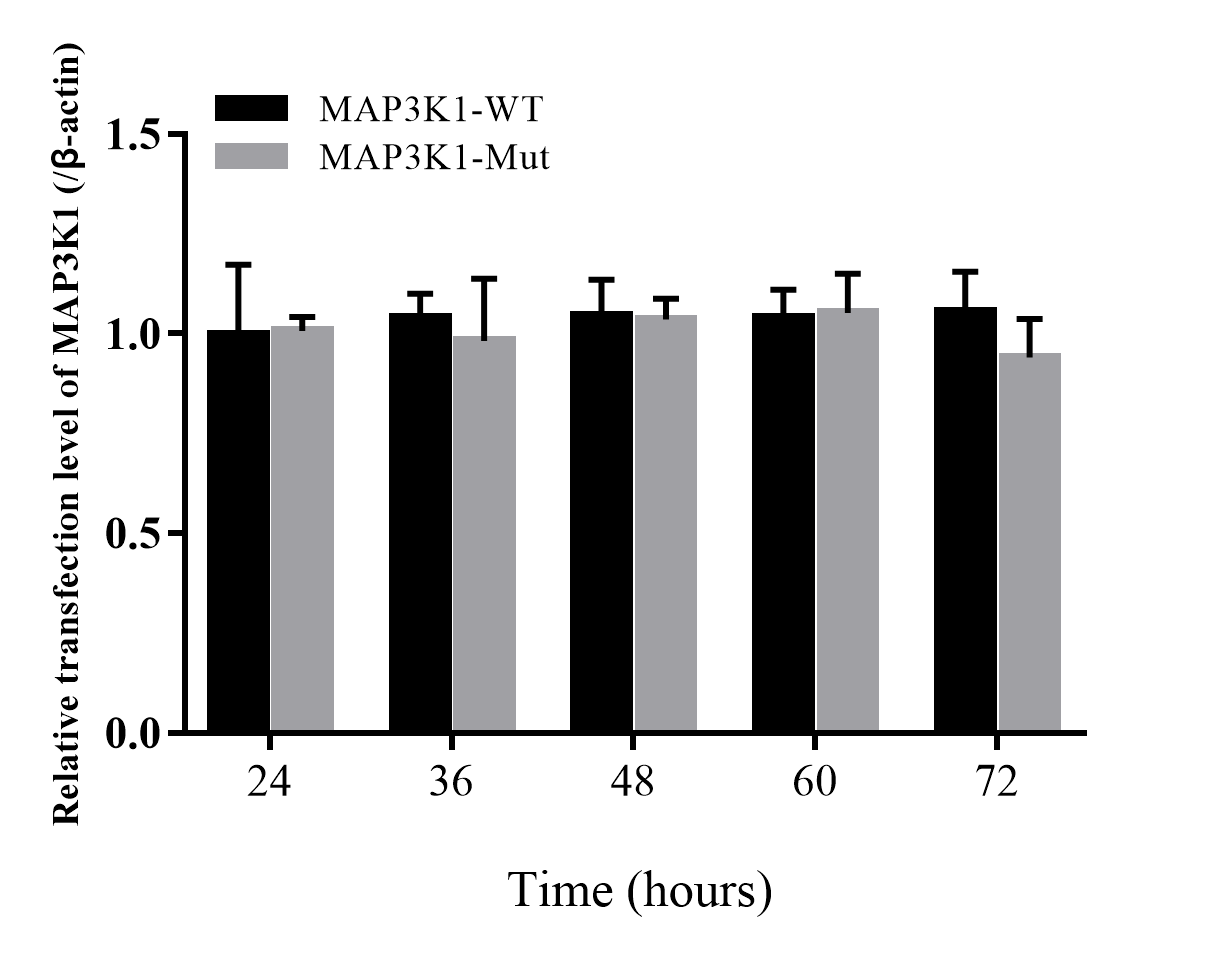

Supplement: Supplementary file 3 [file Image4.TIF]

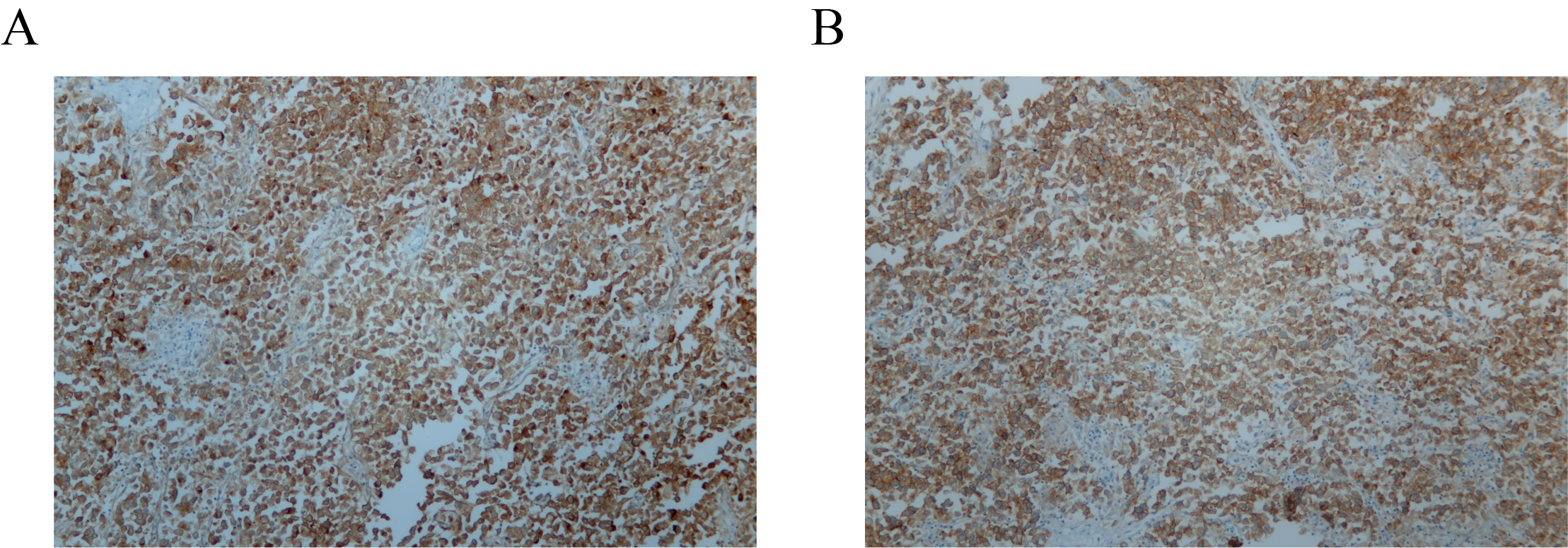

Supplement: Supplementary file 4 [file Image2.TIF]

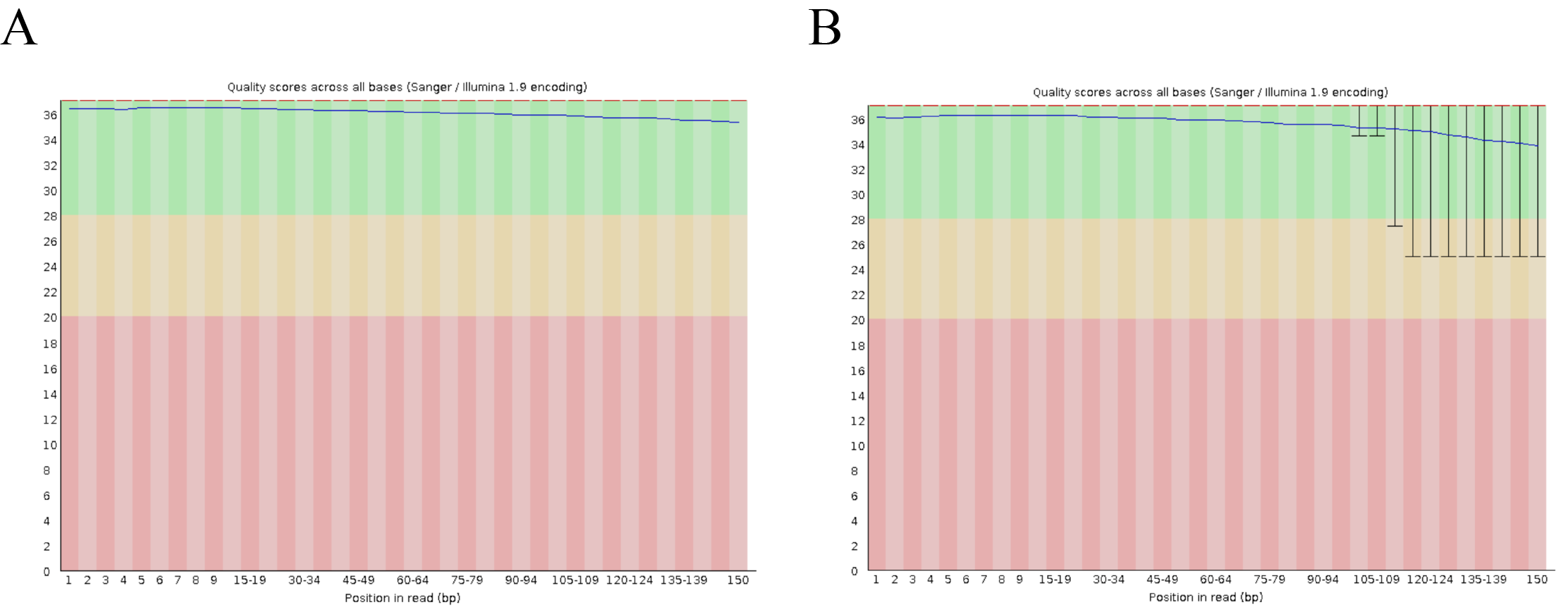

Supplement: Supplementary file 5 [file Image1.TIF]

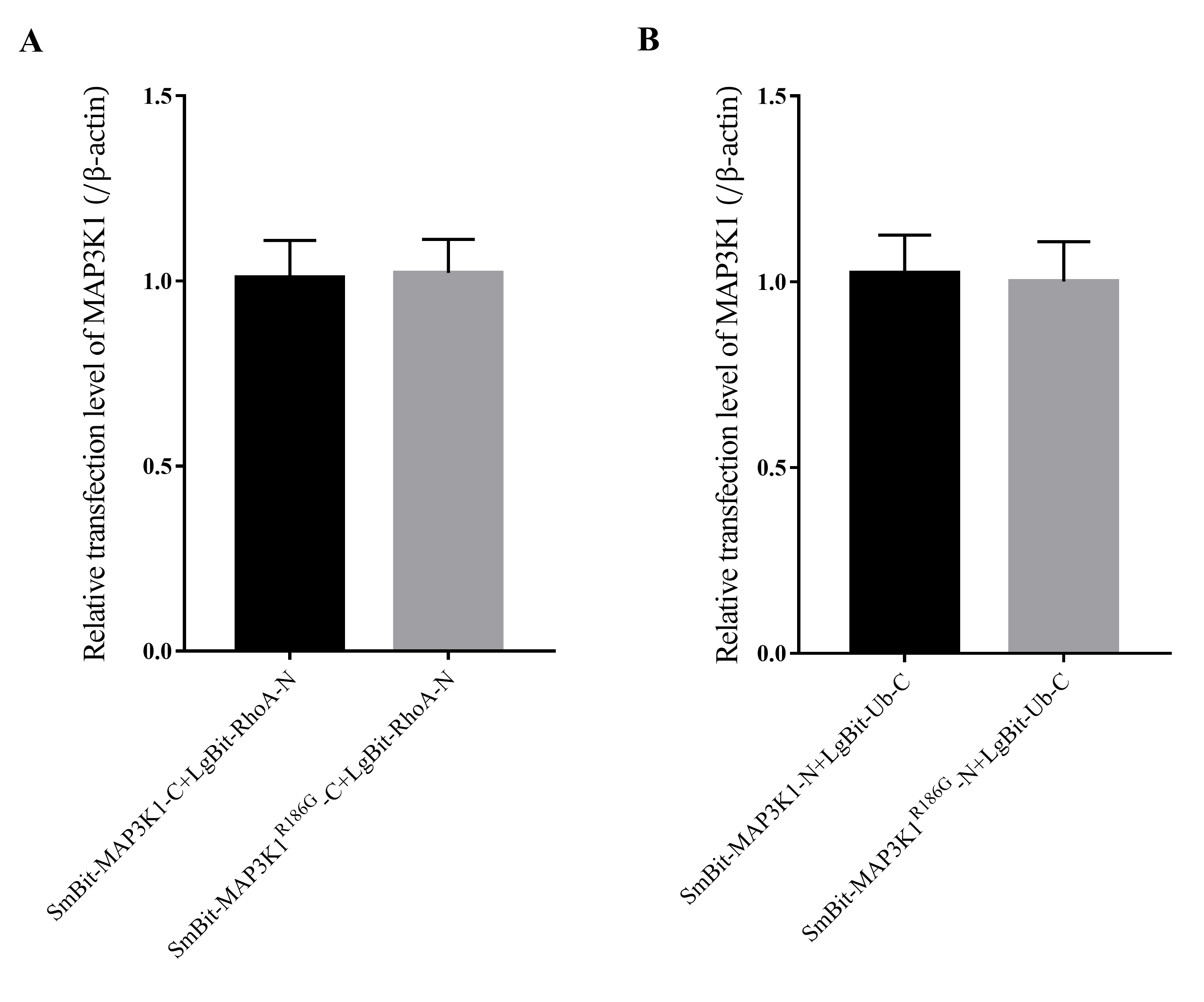

Supplement: Supplementary file 7 [file Image5.TIF]
