# Supplementary material for: MAP3K1 Variant Causes Hyperactivation of Wnt4/β-Catenin/FOXL2 Signaling Contributing to 46,XY Disorders/Differences of Sex Development
Source: Front Genet. 2022 Mar 3;13:736988. doi: 10.3389/fgene.2022.736988 (PMC8927045; doi:10.3389/fgene.2022.736988)
Supplement: Supplementary file 6 [file Table2.DOCX]

**Table S2. Sources and catalogue numbers of antibodies for western blot**

| **Names of antibodies** | **Companies** | **Catalogue numbers** |
| --- | --- | --- |
| α-Tublin | Abcam | ab52866 |
| GSK3β | Cell Signaling | #12456 |
| Phospho-GSK3β (Thr390) | Cell Signaling | #3548 |
| P38 | Cell Signaling | #9212 |
| Phospho-p38 | Cell Signaling | #9215 |
| MAP3K1 | Abclone | A16057 |
| β-catenin | Cell Signaling | #8480 |
| WNT4 | Abcam | ab91226 |
| FOXL2 | Abcam | ab188584 |
| FGFR2 | Cell Signaling | #23328 |
| FGF9 | Abcam | ab223543 |
